# Overexpressing *CYP71Z2* Enhances Resistance to Bacterial Blight by Suppressing Auxin Biosynthesis in Rice

**DOI:** 10.1371/journal.pone.0119867

**Published:** 2015-03-18

**Authors:** Wenqi Li, Fangquan Wang, Jun Wang, Fangjun Fan, Jinyan Zhu, Jie Yang, Fengquan Liu, Weigong Zhong

**Affiliations:** 1 Institute of Food Crops, Jiangsu Academy of Agricultural Sciences/Nanjing Branch of Chinese National Center for Rice Improvement/Jiangsu High Quality Rice R&D Center, Nanjing 210014, China; 2 Institute of Plant Protection, Jiangsu Academy of Agricultural Sciences, Nanjing 210014, China; Shanghai Jiao Tong University, CHINA

## Abstract

**Background:**

The hormone auxin plays an important role not only in the growth and development of rice, but also in its defense responses. We’ve previously shown that the P450 gene *CYP71Z2* enhances disease resistance to pathogens through regulation of phytoalexin biosynthesis in rice, though it remains unclear if auxin is involved in this process or not.

**Methodology and Principal Findings:**

The expression of *CYP71Z2* was induced by *Xanthomonas oryzae* pv. *oryzae* (*Xoo*) inoculation was analyzed by qRT-PCR, with GUS histochemical staining showing that *CYP71Z2* expression was limited to roots, blades and nodes. Overexpression of *CYP71Z2* in rice durably and stably increased resistance to *Xoo*, though no significant difference in disease resistance was detected between *CYP71Z2*-RNA interference (RNAi) rice and wild-type. Moreover, IAA concentration was determined using the HPLC/electrospray ionization/tandem mass spectrometry system. The accumulation of IAA was significantly reduced in *CYP71Z2*-overexpressing rice regardless of whether plants were inoculated or not, whereas it was unaffected in *CYP71Z2*-RNAi rice. Furthermore, the expression of genes related to IAA, expansin and SA/JA signaling pathways was suppressed in *CYP71Z2*-overexpressing rice with or without inoculation.

**Conclusions and Significance:**

These results suggest that *CYP71Z2*-mediated resistance to *Xoo* may be via suppression of IAA signaling in rice. Our studies also provide comprehensive insight into molecular mechanism of resistance to *Xoo* mediated by IAA in rice. Moreover, an available approach for understanding the P450 gene functions in interaction between rice and pathogens has been provided.

## Introduction

Bacterial blight is an important disease in rice caused by *Xoo* that results in severe loss of rice yield worldwide [1]. Rice has evolved to utilize a network of sophisticated signaling pathways against invasion by phytopathogens, for example pathogen-associated molecular patterns (PAMPs), systemic acquired resistance (SAR) and hypersensitive response [2–5]. Plant hormones such as SA, JA and IAA mediate broad-spectrum disease resistance in rice and have been widely studied; the mechanisms of resistance have been elucidated [6, 7].

IAA, the major form of auxin in rice, is generally believed to play an important role in plant growth and development [8, 9]. However, recent studies demonstrate that IAA acts as a negative regulator in the plant immune response [7, 10, 11], as exogenous application of IAA or auxin analogs in rice and *Arabidopsis* significantly promotes disease symptoms. Treatment with IAA and 2,4-dichlorophenoxyacetic acid (2, 4-D; an analog of IAA) in rice resistant to various types of bacterial blight significantly stimulates phytopathogenic *Xoo* proliferation, resulting in high susceptibility to these compounds [12]. Similarly, treatment of resistant rice plants with IAA enhances the infectivity of *Xanthomonas oryzae* pv. *oryzicola* (*Xooc*) and *Magnaporthe oryzae* to rice [13]. In addition, exogenous application of 1-naphthalacetic acid (NAA) or 2,4-D on *Arabidopsis* accelerates the development of disease symptoms during infection by *Pseudomonas syringae* pv. *tomato* (*Pto*) DC3000 or *Pseudomonas syringae* pv. *maculicola* [14, 15].

On the other hand, many phytopathogens are capable of inducing significant IAA accumulation that weakens the host’s native defense barrier, the cell wall [16–20]. This inhibits accumulation of endogenous auxin, leading to high disease resistance rates in rice. The mechanism for this is largely believed to be due to inhibition of expansin gene expression, which induces overexpression of GH3 family genes *OsGH3*.*1*, *GH3-2* and *GH3-8* that enhance broad-spectrum resistance to bacterial *Xoo*, *Xooc* and *Magnaporthe grisea* [12, 13, 21]. Based on these studies, it can be concluded that the suppression of the auxin signaling pathway partly contributes to disease resistance in rice.

The synthesis of IAA is dependent on whether the precursor tryptophan (Trp) is involved, with Trp-dependent and Trp-independent pathways found in both the monocotyledonous model rice and the dicotyledonous model *Arabidopsis* [20, 22, 23]. In Trp-dependent pathways, indole 3-acetaldoxime (IAOx) is one of the key intermediate metabolites [22, 24]. IAOx is a common precursor of auxin, camalexin and indole glucosinolates biosynthesis, and is a crucial branching point from primary metabolism to secondary metabolism in *Arabidopsis* [25–28]. The cytochrome P450 monooxygenase CYP79B2 is responsible for catalyzing the conversion of tryptophan to IAOx in *Arabidopsis* [24, 25, 29, 30]. To date, many other cytochrome P450 monooxygenase genes involved in IAOx biosynthesis and metabolism have been cloned in *Arabidopsis*. The overexpression of *cyp79B2* in *Arabidopsis* significantly increases IAA content [31], though less IAA is detected in the *cyp79B2*/*cyp79B3* double mutant [31]. Another cytochrome P450 monooxygenase, CYP71A13, is capable of catalyzing IAOx to indole-3-acetonitrile (IAN) in the Trp-dependent IAA biosynthesis pathway [32]. P450 monooxygenases CYP83A1 and CYP83B1 have similar biochemical functions for IAA biosynthesis, which maintains the endogenous IAA balance in *Arabidopsis* [33–35]. Unfortunately, both IAO_X_ and IAN have not been detected in rice, though indole-3-acetamide (IAM) is present [36, 37], thus we hypothesize that *Arabidopsis* and rice have different IAA biosynthesis pathways.

The pathway for IAA biosynthesis is very complex in rice, though a few genes involved in IAA signaling have been cloned, including the *YUCCA* family, the SMALL AUXIN-UP RNA (*SAUR*) family, the *GH3* family and the AUXIN/INDOLE-3-ACETIC ACID (*Aux/IAA*) family [38–42]. The cytochrome P450 family is the largest enzymatic protein family in rice and is largely responsible for both growth and development and the defense response [43–47]. In total, 356 P450 genes and 99 related pseudogenes have been identified in rice (indica and japonica) genomes using sequence information [48]. However, it remains unclear whether P450 genes involved in disease resistance to *Xoo* are responsible for regulating the IAA signaling pathway in rice.

A previous study by our group showed that the cytochrome P450 gene *CYP71Z2* contributes to bacterial blight resistance by mediating diterpenoid phytoalexin accumulation in rice [47]. Here we present studies on the role of *CYP71Z2* in auxin signaling pathway.

## Materials and Methods

### Constructs and transformation

To construct the *CYP71Z2* promoter GUS reporter vector, the predicted 1098 bp DNA fragment upstream of the start codon was amplified from Nipponbare genomic DNA and then inserted into the binary expression vector pBI121. The primers used in this study are shown in S2 Table. The recombinant plasmids were transformed into *Agrobacterium tumefaciens* strain EHA105 using a freeze-thaw method. Subsequently, the T-DNA region with the predicted *CYP71Z2* promoter was introduced into calli derived from mature Nipponbare embryos using the *Agrobacterium*-mediated method [47].

### Plant materials and growth conditions

Three seedlings overexpressing *CYP71Z2* and different RNAi-expressing rice were chosen to analyze the role of IAA in *Xoo* resistance. Transgenic seedlings (T5, T6 and T7) were grown in a growth chamber (12 h photoperiod; 28°C; 70% relative humidity; light strength, 30,000 lx), and a slow-release fertilizer was applied. All plants (wild-type and transgenic) were then transplanted into pots under normal growth conditions for *Xoo* inoculation, IAA quantification, RNA extraction and harvest.

### Measurement of disease resistance

Resistance of rice to the bacterial blight pathogen Philippine *Xoo* strain PXO99^A^ was evaluated by the leaf-clipping method at the booting stage. Level of resistance was classified into six groups and measured using the percentage of diseased area (lesion length/leaf length) at 2–3 weeks following inoculation. The six classifications are: 1) Leaves without obvious lesions have high resistance, 2) Leaves with diseased area less than 10% have resistance, 3) Leaves with diseased area ≥ 10% but < 20% have modest resistance, 4) Leaves with diseased area ≥ 20% and < 50%, have modest susceptibility, 5) Leaves with diseased area ≥ 50% and < 75% have susceptibility and 6) Leaves with diseased area ≥ 75% have high susceptibility [47]. The bacterial growth rate in rice leaves was determined by counting colony-forming units [47].

### Bioinformatic analysis of *CYP71Z2*

The *CYP71Z2* promoter region was predicted using the online Promoter Scan (http://www-bimas.cit.nih.gov/molbio/proscan/). The cis-acting regulatory DNA elements of the *CYP71Z2* promoter were determined by searching in the PLACE (http://www.dna.affrc.go.jp/PLACE/signalscan.html) database. Phylogenetic analysis among eight species was performed by MEGA. Protein sequence alignment was performed by searching in the non-redundant protein sequences database of the NCBI.

### Gene expression analyses

Leaf samples next to the sites of bacterial infection were collected for RNA extraction at different time points post-inoculation with *Xoo* PXO99^A^. Quantitative real-time PCR (qRT-PCR) was conducted on the Applied Biosystems 7500 real-time PCR system using SYBR Premix Ex Taq^TM^ according to the manufacturer’s instructions. For qRT-PCR analysis, three independent biological samples were used, each with three technical replicates. The internal reference gene *EF-1a* (accession no. AK061464) was used to standardize RNA quantities. Primers used in this study for qRT-PCR are shown in S2 Table.

### GUS histochemical staining and protein activity

Rice tissue, including leaf, root and stem, were put into GUS staining solution for ∼5 hours at 37°C. The staining solution was removed and 75% alcohol was added to wash off excess stain, as described previously [12]. After complete decolorization, photographs of the stained rice tissue were examined using electron microscopy.

### IAA quantification

To determine the concentration of IAA in rice, at least 1 g of sample was cut from each leaf at different time points post-inoculation and kept frozen at −80°C until use. IAA measurement conditions are described in the methods of [12]. IAA concentration was determined using the HPLC/electrospray ionization/tandem mass spectrometry system and the peaks of the precursor ions 176.3 after purification with a C18-SepPak cartridge.

## Results

### Phylogenetic analysis of *CYP71Z2*

**Fig 1:**
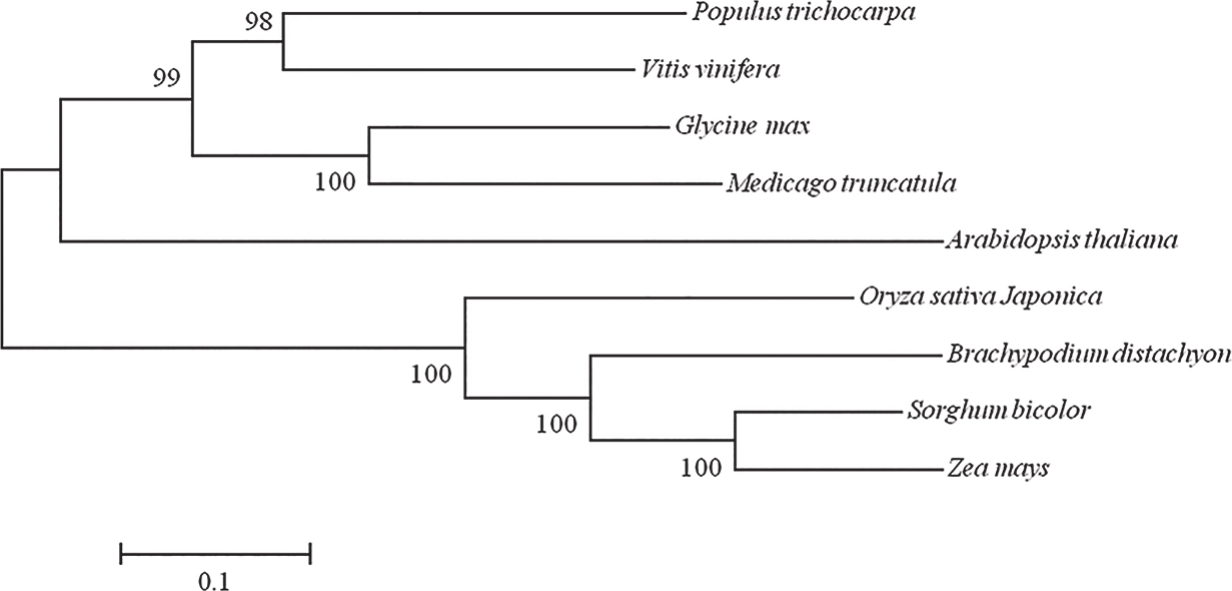
Phylogenetic relationship among the CYP71Z2 homologues in plants. Amino acid sequences of the CYP71Z2 homologues were obtained from the NCBI Genbank. The scale bar indicates the number of amino acid substitutions per site. The numbers at the nodes indicate the level of bootstrap support.

Our previous study showed that *CYP71Z2*, a cytochrome P450 gene involved in biosynthesis of diterpenoid phytoalexin, plays a role in resistance to bacterial blight in rice [47]. Other P450 genes, like *cyp83B1*, *cyp79B2* and *cyp79B3*, are also known to play important roles for auxin metabolites in plants [31, 33]. Sequence alignment suggests that the amino acid sequence of CYP71Z2 shows similarity to the following proteins in various plants: AT3G26210 (46.4% identity, *Arabidopsis thaliana*, CYP71B23), LOC100824609 (62.1% identity, *Brachypodium distachyon*, CYP71D7-like), Sb01g020810 (61.7% identity, *Sorghum bicolor*), LOC100273457 (61.7% identity, *Zea mays*), 7467291 (50.3% identity, *Populus trichocarpa*, CYP71D26), LOC100794503 (50.2% identity, *Glycine max*), MTR-5g018990 (46.2% identity, *Medicago truncatula*) and LOC100263449 (46.0% identity, *Vitis vinifera*). However, no similarity was observed between amino acid sequences of CYP71Z2 and known P450s CYP83B1, CYP79B2 and CYP79B3. Further, a phylogenetic tree was constructed using MEGA (Molecular evolutional genetics analysis) and the eight homologous proteins having high identity with CYP71Z2 (Fig. 1). Considering the bioinformatic data, our results rationalize studying the function of the P450 gene *CYP71Z2* in rice.

### The expression patterns of *CYP71Z2*

The predicted promoter region of *CYP71Z2* was determined by using Promoter Scan (http://www-bimas.cit.nih.gov/molbio/proscan/) and PLACE (http://www.dna.affrc.go.jp/PLACE/). The TATA-box is located at 535 bp, and the DNA fragment of 301–551 bp is the predicted promoter region. The promoter sequence also contains *cis*-acting elements (CAAT-box, W-box, MYB, ASF-1, etc. binding motifs) involved in salicylic acid, auxin, photo-responsive and flavonoid biosynthesis processes.

**Fig 2:**
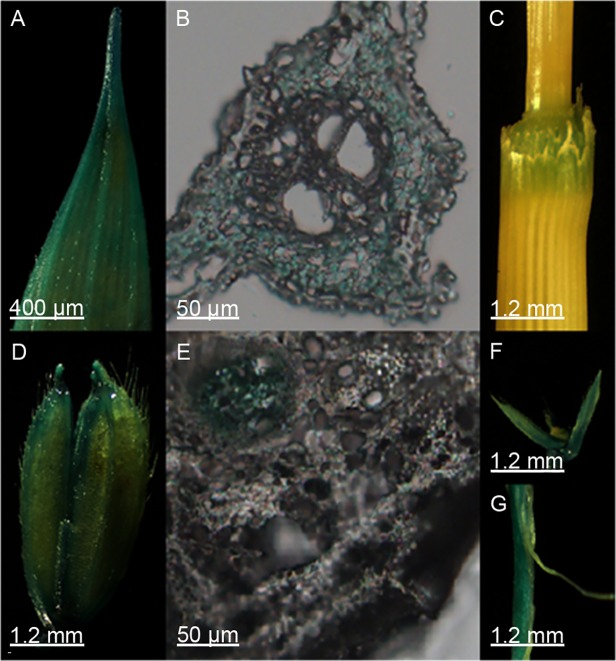
Expression patterns of GUS driven by the *CYP71Z2* promoter in various organs and tissues of transgenic rice plant. Shown are leaf **(A)**, node **(C)**, lemma and palea **(D, F)**, primary root **(G)** and transverse section of a leaf **(B)** and node **(E)**. Scale bars are 400 μm **(A)**, 50 μm **(B, E)** and 1.2 mm **(C**, **D**, **F** and **G)**.

**Fig 3:**
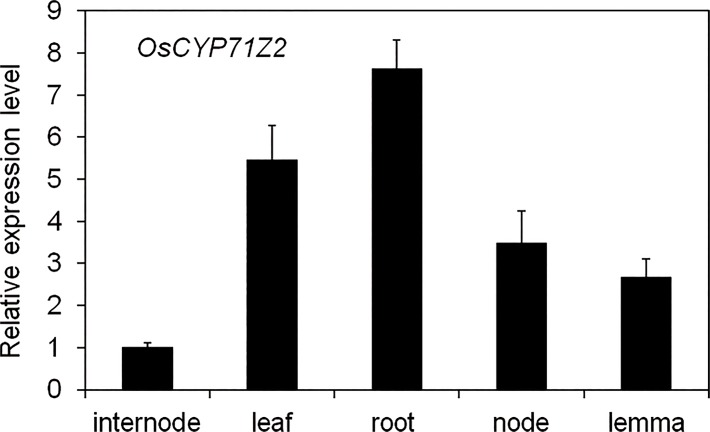
Expression levels of *CYP71Z2* in leaf, root, node, internode and lemma. Data represent means of three replicates ± standard deviation.

To assess the expression pattern of *CYP71Z2*, a *CYP71Z2* promoter/GUS protein (*β*-glucuronidase) fusion expression vector was constructed (S1 Fig.). *CYP71Z2* promoter/*GUS* transgenic plants were generated by transforming *japonica* cultivar Nipponbare. All seven confirmed transgenic lines showed a common pattern of GUS distribution, although differences were observed in GUS activity. GUS histochemical staining showed *CYP71Z2* mainly expressed in the leaves, node, lemma, palea and primary roots, indicating tissue-specific expression patterns of *CYP71Z2* in rice (Fig. 2). Gene expression patterns were analyzed by qRT-PCR, which showed high expression in the leaves, root node and internodes, consistent with results from GUS histochemical staining (Fig. 3).

**Fig 4:**
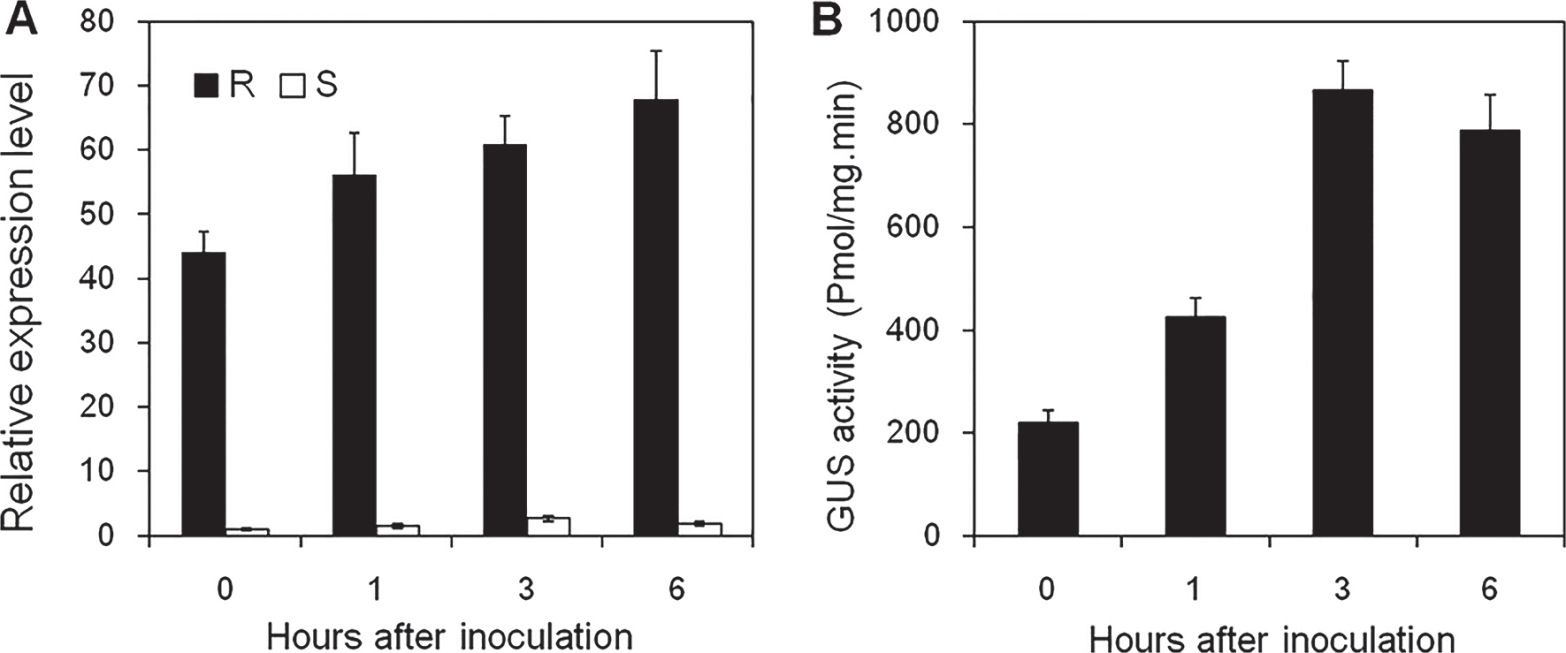
Expression of *CYP71Z2* was induced in *Xoo* resistant rice cultivar upon *Xoo* infection. (A) Rice cultivar resistance to *Xoo* had a much higher basal expression level of *CYP71Z2* than rice cultivar susceptible to *Xoo*, and showed an induced expression level of *CYP71Z2* after inoculation with *Xoo* PXO99^A^. R, *Xoo* resistant rice cultivar NJH12. S, *Xoo* susceptible rice cultivar R109. (B) An increased GUS activity was observed in transgenic rice plants harbouring the P_*CYP71Z2*_::GUS construct after inoculation of *Xoo*.

Expression patterns of *CYP71Z2* during incompatible and compatible interactions between rice and bacterial blight were detected by qRT-PCR, with results showing that expression of *CYP71Z2* in resistant rice NJH12 was higher than that in susceptible rice R109 (Fig. 4A). In addition, GUS activity in the leaves of *CYP71Z2* promoter-driven transgenic rice plants significantly increased after inoculation with *Xoo* (Fig. 4B). These results suggest that *CYP71Z2* is quickly activated in rice upon infection with the bacterial blight pathogen *Xoo*.

### Overexpressing *CYP71Z2* increases resistance to *Xoo*

**Fig 5:**
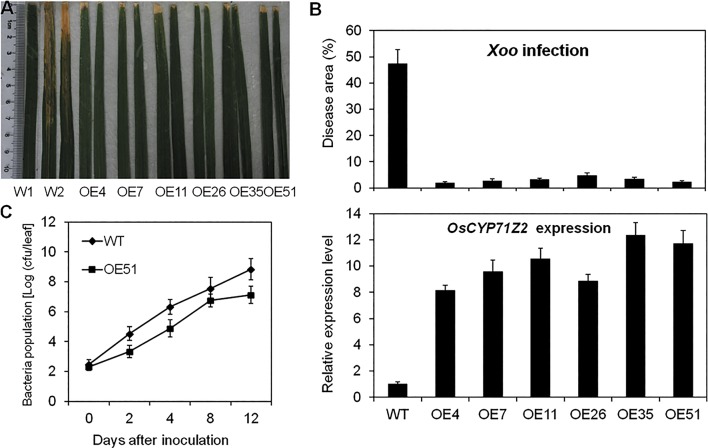
Increased resistance to *Xoo* in *CYP71Z2*-overexpressing lines. (A) *CYP71Z2*-overexpressing lines (T_7_) showed enhanced resistance to *Xoo* strain PXO99^A^. (B) Overexpression of *CYP71Z2* was positively correlated with suppression of disease development. *CYP71Z2* expression was analyzed by qRT-PCR. Data represent means of three replicates ± standard deviation. (C) Growth of *Xoo* strain PXO99^A^ in leaves of rice plants overexpressing *CYP71Z2* was suppressed. W1, Wild-type Nipponbare inoculated with water. W2, Wild-type Nipponbare inoculated with *Xoo*. cfu, colony-forming unit. Data represent means of three replicates ± standard deviation.

Previous studies have shown that the rice P450 gene *CYP71Z2* is involved in diterpenoid phytoalexin biosynthesis, contributing to bacterial blight resistance [47]. In this study, we selected six *CYP71Z2*-overexpressing rice (Acceptor rice is the Nipponbare; T5, T6 and T7) to identify the role of auxin in *CYP71Z2*-mediated blight resistance at the booting stage. Six homozygous *CYP71Z2*-overexpressing lines showed resistance to *Xoo* strain PXO99^A^, with the average lesion area ranging from 1.86–4.75%, compared with an average of 47.37% in wild-type Nipponbare (Fig. 5A, B; S1 Table). The expression of *CYP71Z2* in all six T7 *CYP71Z2*-overexpressing plants was higher than that in wild-type, showing increases of approximately 8.16- to 12.35-fold (Fig. 5B; S1 Table). Furthermore, bacterial growth analysis showed that the growth rate of PXO99^A^ in T7 *CYP71Z2*-overexpressing line OE51 was lower than that in wild-type after inoculation (Fig. 5C). These results suggest that overexpression of *CYP71Z2* confers rice with durable, stable resistance to *Xoo*.

**Fig 6:**
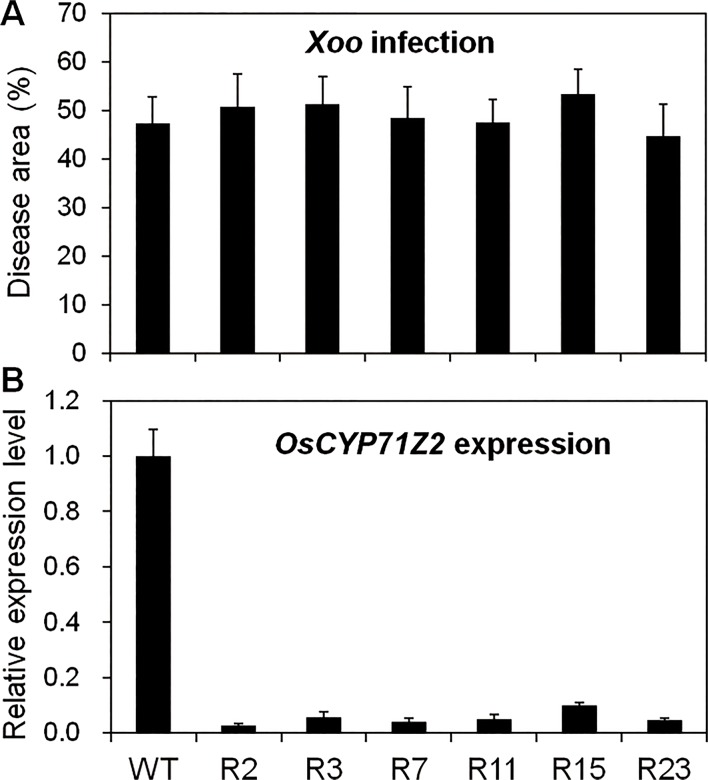
Knock-down *CYP71Z2* had no impact on *Xoo* resistance. (A) No significant difference in disease development was observed between *CYP71Z2*-RNAi lines and wild-type in response to inoculation of *Xoo* strain PXO99^A^. (B) Expression levels of *CYP71Z2* in *CYP71Z2*-RNAi lines and wild-type. Data represent means of three replicates ± standard deviation.

We also examined resistance to *Xoo* in *CYP71Z2*-RNAi lines (T5, T6 and T7) at the booting stage. Expression of *CYP71Z2* in RNAi lines was significantly reduced, as shown in Fig. 6. Moreover, none of the RNAi lines showed a significant difference in response to PXO99^A^ infection compared with wild-type (Fig. 6; S1 Table). These results show that suppression of *CYP71Z2* expression does not significantly increase susceptibility to PXO99^A^ in *CYP71Z2*-RNAi transgenic lines, suggesting that functional redundancy among the CYP71Z of family may mask the effect of *CYP71Z2*.

### *CYP71Z2* negatively regulates IAA metabolism

**Fig 7:**
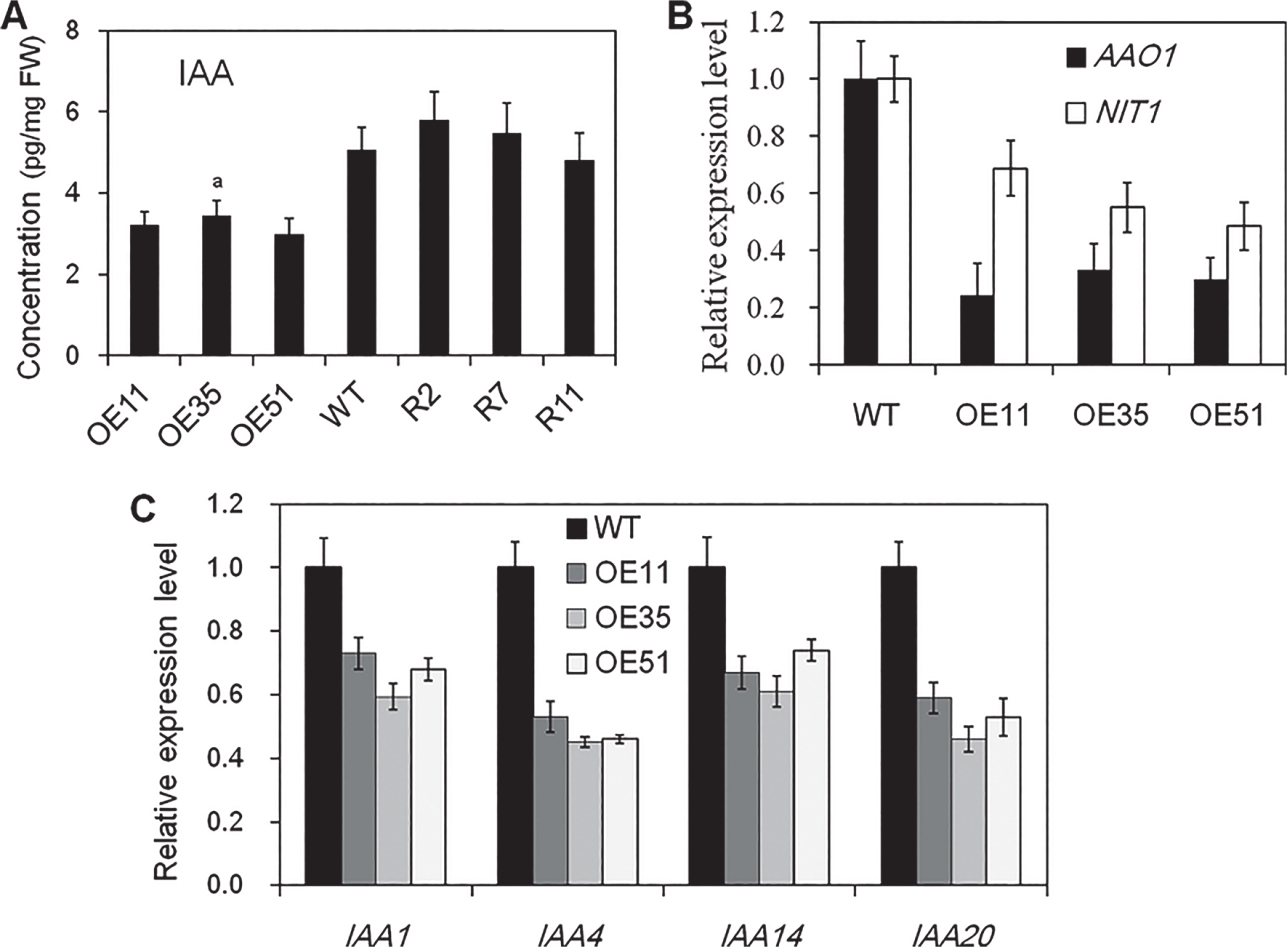
Overexpression of *CYP71Z2* suppressed accumulation of endogenous IAA and the expression levels of genes related to auxin biosynthesis and signaling. (A) Quantification of free IAA content in the leaves of *CYP71Z2*-overexpressing rice plants at the ripening stage. (B) Expression patterns of genes related to auxin biosynthesis in *CYP71Z2*-overexpressing rice plants. (C) Expression patterns of auxin-responsive genes in *CYP71Z2*-overexpressing rice plants. Data represent means of three replicates ± standard deviation. a indicates that a significant difference (*P* < 0.05) was detected between *CYP71Z2*-overexpressing rice and wild-type plants. WT, wild-type Nipponbare; FW, fresh weight.

The endogenous phytohormone auxin is known to be involved in resistance of rice to bacterial blight [12], though it is unclear if auxin is involved in signaling pathways that contribute to *CYP71Z2*-mediated blight resistance. To examine this possibility, we measured free IAA concentration of *CYP71Z2* transgenic lines at the booting stage. The concentration of endogenous free IAA in the *CYP71Z2*-overexpressing lines OE11, OE35 and OE51 was 3.21, 2.99 and 3.64 pg/mg fresh leaves, respectively. Compared with 5.07 pg/mg fresh leaves in wild-type, these results suggest that IAA expression is 1.47- to 1.7-fold lower than that of wild-type plants (Fig. 7A), which likely contributes to the resistance to *Xoo* of these transgenic plants. Moreover, the endogenous free IAA levels in the leaves of *CYP71Z2*-RNAi lines showed no significantly differences compared to those of wild-type (Fig. 7A), supporting the hypothesis of functional redundancy among CYP71Z family proteins in rice.

To further analyze whether decreased IAA in *CYP71Z2*-overexpressing lines was caused by other genes of the IAA biosynthesis pathway, we quantified the expression of *AAO1* (indole-3-acetaldehyde oxidase) and *NIT1* (nitrilase) in rice using qRT-PCR. The sequence alignment showed 72% and 78% identity with homologous genes *AAO1* and *NIT1*, respectively, in *Arabidopsis* [12]. Previous reports indicated that *AAO1* and *NIT1* function in two Trp-dependent IAA biosynthesis pathways (indole-3-pyruvic acid and indole-3-acetaldoxime) [12, 38]. Quantitative analysis showed that expression of *AAO1* and *NIT1* in OE11, OE35 and OE51 *CYP71Z2*–overexpressing plants was lower by 3.01 to 4.12-fold and 1.46 to 2.01-fold, respectively, than that in wild-type (Fig. 7B). These results support the notion that *CYP71Z2* negatively regulates *AAO1* and *NIT1* expression to suppress IAA accumulation.

In addition, previous reports demonstrated that auxin signaling is also affected by changes in IAA concentration [12]. To evaluate this in our study, we analyzed the expression of auxin signaling-related genes (*Aux/IAA* families) in *CYP71Z2*–overexpressing lines by qRT-PCR. These results show that expression of *IAA1*, *IAA4*, *IAA14* and *IAA20* was lower in *CYP71Z2*–overexpressing lines (OE11, OE35 and OE51) than in wild-type, especially with respect to *IAA4* and *IAA20* (Fig. 7C).

### IAA biosynthesis is suppressed in *CYP71Z2*–overexpressing rice after inoculation with *Xoo*

**Fig 8:**
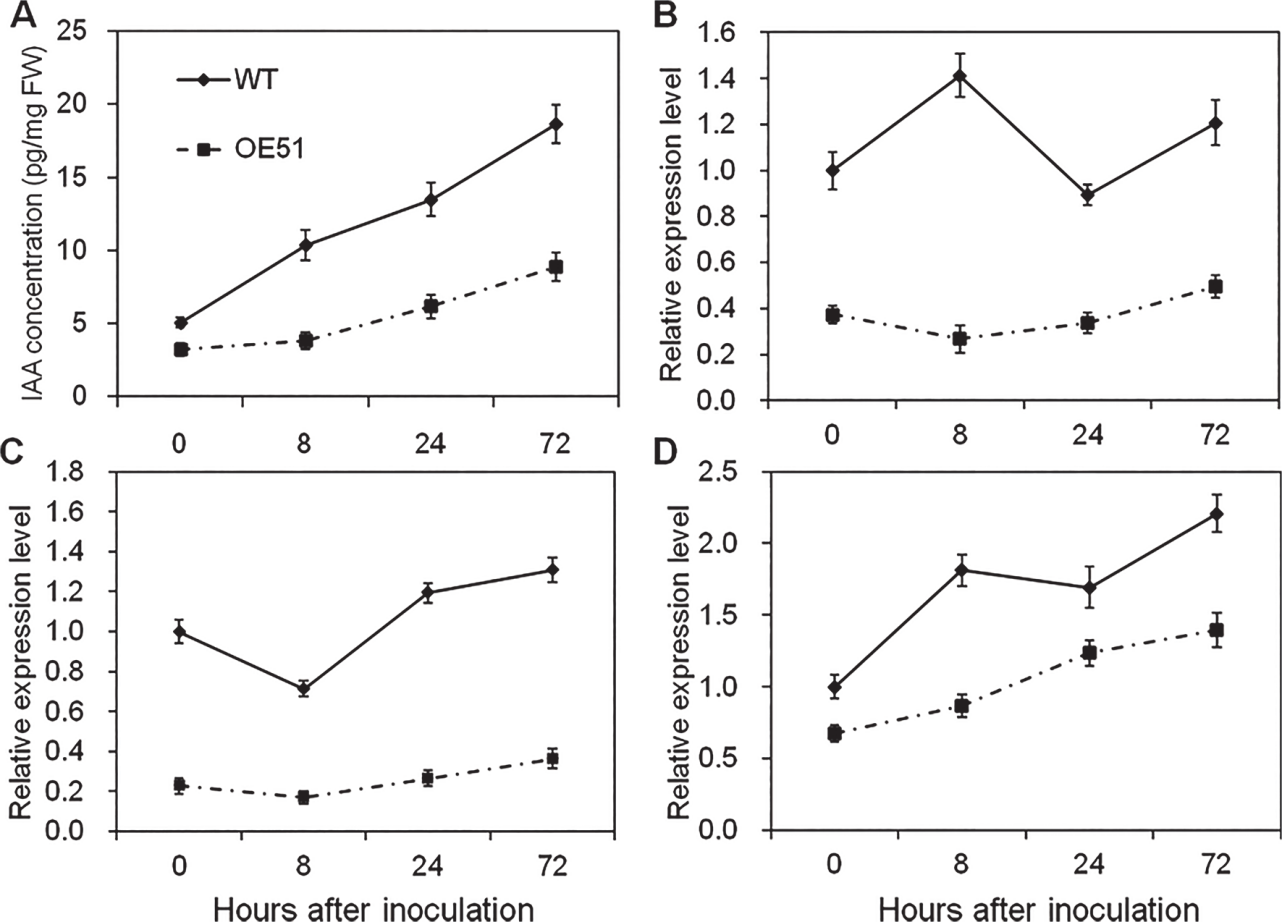
Overexpression of *CYP71Z2* suppressed the IAA signaling pathway in rice after inoculation with PXO99^A^. (A) Quantification of free IAA in the leaves of *CYP71Z2*-overexpressing rice after inoculation at the ripening stage. Transcript levels of genes *AAO1* (B), *IAA1* (C) and *IAA20* (D) in *CYP71Z2*-overexpressing rice after inoculation were determined by qRT-PCR. Data represent means of three replicates ± standard deviation.

Previous reports demonstrated that auxin signaling is involved in rice-*Xoo* interactions, with auxin seeming to act as a negative regulator of resistance to *Xoo* in rice [10–12]. To further examine whether auxin signaling takes part in disease resistance to *Xoo*, we analyzed the IAA concentration in the *CYP71Z2*–overexpressing rice OE51 after inoculation with *Xoo* strain PXO99^A^. As shown in Fig. 8A, accumulation of IAA was induced at 8, 24 and 72 hours post-inoculation in both OE51 and wild-type. However, IAA accumulation in OE51 was found to be significantly lower than that in wild-type regardless of whether the plants were inoculated or not, with up to a 2.7-fold decrease in accumulation at 8 hours after innoculation (Fig. 8A). These results suggest that overexpression of *CYP71Z2* in rice negatively regulates IAA biosynthesis in response to *Xoo* infection.

The expression of auxin signaling and biosynthetic genes was also analyzed by qRT-PCR in the resistant transgenic OE51 and the susceptible wild-type rice following *Xoo* inoculation. Expression of *AAO1*, *IAA1* and *IAA20* was induced in both OE51 and wild-type plants after infection, though expression of *AAO1* and *IAA20* was significantly decreased in wild-type at 24 h post-inoculation (Fig. 8B, C and D). However, we found that expression of *AAO1*, *IAA1* and *IAA20* in OE51 was significantly suppressed compared with wild-type after inoculation (Fig. 8B, C and D), suggesting that overexpression of *CYP71Z2* in rice suppresses the expression of genes involved in auxin signaling.

### Overexpressing *CYP71Z2* inhibits expression of expansin genes

**Fig 9:**
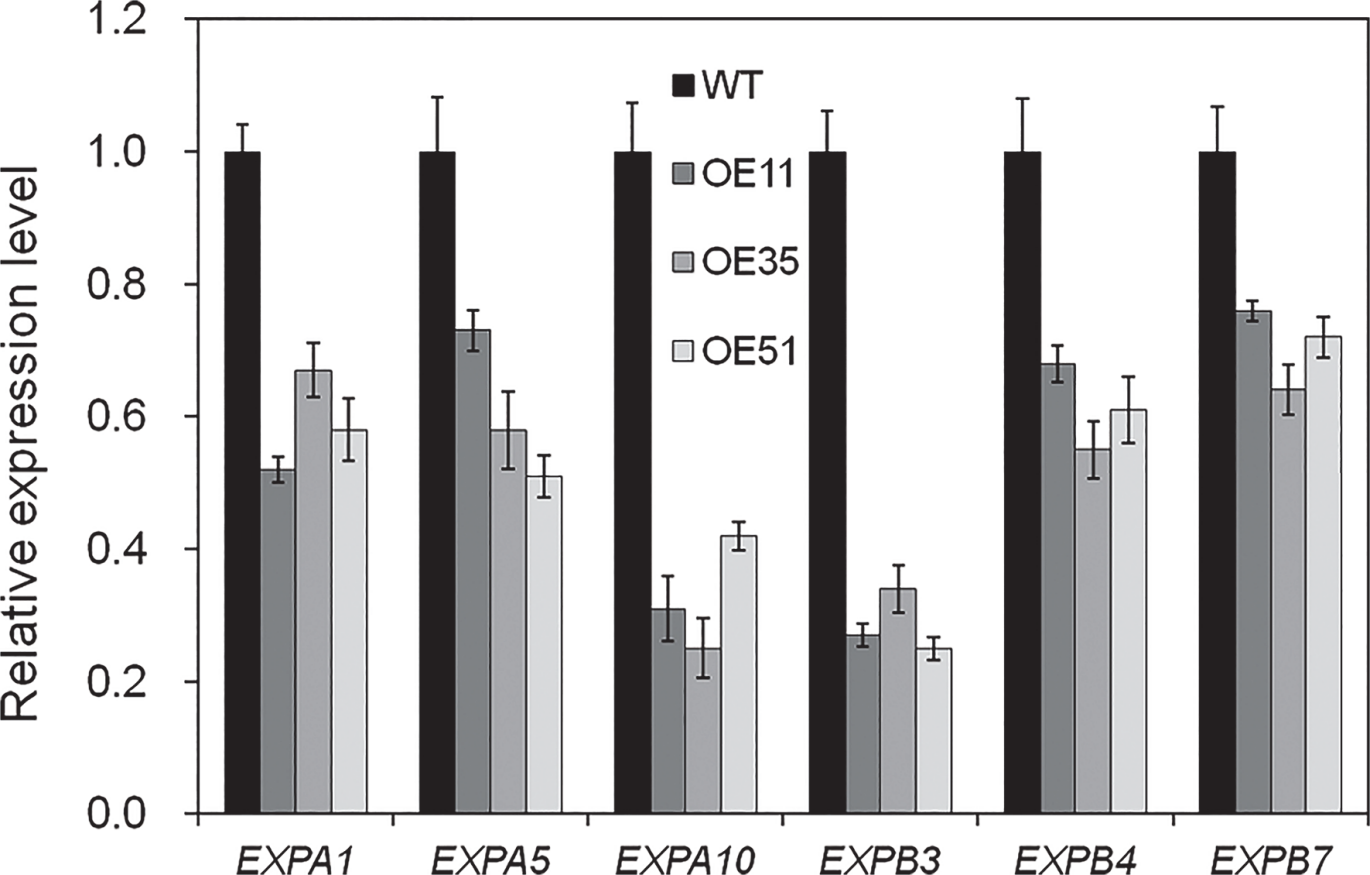
Overexpression of *CYP71Z2* had a negative impact on the expression of expansin genes. Data represent means of three replicates ± standard deviation. WT, wild-type Nipponbare.

The plant cell wall is a natural protective barrier for phytopathogens. The loosening cell walls are easier to be infected by pathogenic bacteria. Suppression of expansion genes can prevent plant cell walls from loosening, resulting in enhanced physical protection of plants to phytopathogens [12]. To examine their role in the resistance to *Xoo*, we determined the expression of six expansin genes in *CYP71Z2*-overexpressing rice, including three rice *α*-expansin genes (*EXPA1*, *EXPA5* and *EXPA10)* and three rice *β*-expansin genes (*EXPB3*, *EXPB4* and *EXPB7)*. qRT-PCR analysis showed that expression of all six expansin genes was decreased in *CYP71Z2*-overexpressing rice compared with wild-type under normal growth condition (Fig. 9). These results demonstrate that expansin genes may partly contribute to the resistance to *Xoo* in *CYP71Z2*-overexpressing rice.

### The SA/JA pathway is not involved in *Xoo* resistance of *CYP71Z2*-overexpressing rice

**Fig 10:**
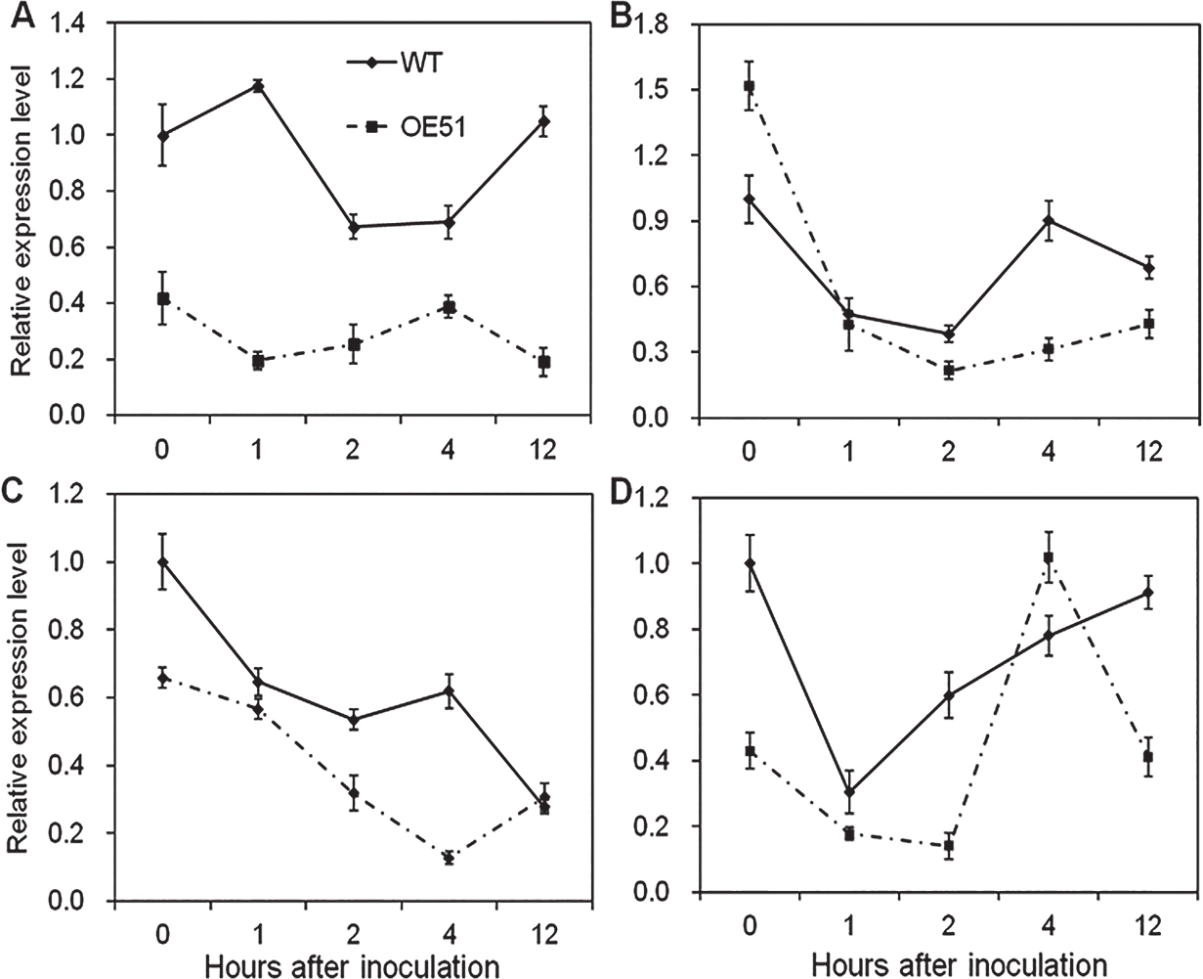
Overexpression of *CYP71Z2* inhibited the expression levels of genes involved in disease resistance pathway mediated by SA/JA. The expression levels of four genes *PR1a* (A), *PR1b* (B), *AOS2* (C) and *LOX* (D) functioning in the SA/JA-dependent disease resistance pathways in *CYP71Z2*-overexpressing rice plant. Data represent means of three replicates ± standard deviation.

Previous studies suggested that SA/JA defense responses are independent of the resistance pathway mediated by auxin in rice [12]. To test whether the increased resistance to *Xoo* in *CYP71Z2*-overexpressing rice accompanied by the inactivation of SA- and JA-dependent defense pathways, we detected transcripts of four key genes (*PR1a*, *PR1b*, *LOX* and *AOS2*) that act in two distinct classes of defense signaling pathways. Relative expression levels analyzed by qRT-PCR showed that three genes had lower expression in OE51 than in wild-type without inoculation, with reductions in *AOS2*, *LOX* and *PR1a* being 1.52-, 2.33- and 2.39-fold, respectively, although *PR1b* was increased by 1.52-fold in OE51 compared with wild-type (Fig. 10). During *Xoo* infection, these four genes were largely suppressed in OE51 at most time points (Fig. 10). Gene expression analysis demonstrated that *CYP71Z2* may function as a negative regulator of the SA/JA defenses signaling pathways during the incompatible interaction between rice and *Xoo*, which is consistent with results shown in a previous study [12].

## Discussion

The mechanisms behind bacterial and fungal disease resistance in rice remain largely unknown, though some *GH3* genes and auxin biosynthesis regulators have been implicated in the process. Our previous study showed that the P450 gene *CYP71Z2* is involved in resistance to *Xoo* infection through activation of the phytoalexin biosynthesis pathway. In this study, we’ve found that overexpression of *CYP71Z2* confers transgenic (T5, T6 and T7) rice with durable, stable resistance to bacterial blight, which is accompanied by up-regulation of genes related to IAA biosynthesis and IAA response pathways. Moreover, no significant differences were observed in resistance to *Xoo* and IAA accumulation between *CYP71Z2*-RNAi and wild-type lines, suggesting some functional redundancy to compensate for reduced *CYP71Z2* expression. These results demonstrate that the cytochrome P450 gene *CYP71Z2* is involved in disease resistance to *Xoo*, potentially through negative regulation of IAA/auxin biosynthesis.

The role of auxin in plant disease resistance has been widely studied, with recent evidence demonstrating IAA’s role as a negative regulator of plant disease resistance to bacterial and fungal pathogens [49, 50]. The role of GH3-like proteins in disease resistance mediated by IAA has also been gradually elucidated over recent years [12, 13, 21]. In this study, homozygous *CYP71Z2*-overexpressing rice showed durable resistance to *Xoo* accompanied by a reduction in IAA accumulation (Fig. 5, 7), consistent with the resistance phenotype of IAA-deficient plants in previous reports [12, 13, 21]. In addition, the putative indole-3-acetaldehyde oxidase (*AAO1*) and nitrilase (*NIT1*), two key genes related to auxin synthesis in rice [12, 38], were found to have reduced expression in *CYP71Z2*-overexpressing rice (Fig. 7). These results further confirm that suppression of auxin biosynthesis contributes to disease resistance of *CYP71Z2*-overexpressing rice, and overall importance of auxin regulation in response to pathogenic infection.

P450 genes have been reported to either act as either positive or negative regulators of auxin homeostasis in *Arabidopsis*. A plausible explanation for this may be that the substrates catalyzed by different P450 oxidases are different, resulting in changes in IAA production. For example, *cyp83B1* mutants show significant overproduction of auxin, whereas *CYP83B1*-overexpressing lines display a loss of apical dominance that is typical of auxin deficiency [33–35]. These studies also showed that the CYP83B1 protein is responsible for converting IAOx to 1-aci-nitro-2-indolyl-ethane, which functions to maintain the dynamic balance between IAA and indole glucosinolate metabolism [34, 35]. In addition, the cytochrome P450 enzyme CYP79B2 catalyzes the transformation of tryptophan into IAOx, playing a positive role in IAA biosynthesis [29, 30]. In this study, we demonstrate that another P450 gene, *CYP71Z2*, shows similar results when overexpressed as the CYP83B1 mutants, suggesting that *CYP71Z2* plays a negative regulatory role in IAA biosynthesis. Unfortunately, substrates for CYP71Z2 and the mechanism behind this role have not yet been identified.

IAOx and IAN are two key intermediates of camalexin metabolism and IAA biosynthesis in *Arabidopsis*, suggesting cross-talk between these two pathways [22, 24]. Overexpression of *CYP79B2* in *Arabidopsis* has been shown to increase IAA content and lead to excessive auxin production, which was likely due to CYP79B2 catalyzing the transformation of tryptophan into IAOx. Interestingly, *CYP79B2*/*CYP79B3* double mutants had reduced levels of both IAA and camalexin, suggesting some degree of similar regulation between the two pathways [24, 32]. Moreover, CYP71A13 was shown to catalyze the conversion of IAOx to IAN, which also led to reductions in IAN and camalexin upon *cyp71A13* mutation [32]. Taken together, these data indicate that cross-talk likely exists between the auxin and camalexin biosynthetic pathways in *Arabidopsis*. Results from our study showing reduced IAA accumulation in *CYP71Z2*-overexpressing rice (Fig. 7A), in conjunction with previous reports showing that *CYP71Z2* accelerates phytoalexin biosynthesis [47], lead us to speculate that cross regulation of IAA and phytoalexin biosynthesis also exists in rice, though this hypothesis requires further study.

Many phytopathogens produce IAA for survival and multiplication during the infection process [16–20]. Pathogen-produced IAA leads to induction of the expression of rice expansin genes, resulting in an increase in long-term cell wall flexibility [12, 51]. This process makes plant cell walls vulnerable and contributes to pathogen infection and multiplication in rice. In this study, the relative expression of expansin genes in *Xoo*-resistant, *CYP71Z2*-overexpressing rice was significantly decreased and correlated with suppression of IAA signaling (Fig. 7–9). These results suggest that the suppression of expansin genes may also contribute to disease resistance in *CYP71Z2*-overexpressing rice, though the mechanisms behind this remain unclear.

As has been shown, auxin biosynthesis is suppressed in resistant rice and is always accompanied by decreases in the expression of auxin-responsive genes. The expression of auxin signaling–related genes was found to be significantly decreased in auxin-deficient, *GH3-8*-overexpressing plants exhibiting resistance to *Xoo* [12, 52, 53]. Consistently, the accumulation of auxin signaling-related genes *AAO1*, *IAA1* and *IAA20* was inhibited in *CYP71Z2*-overexpressing rice (Fig. 8). This suggests that suppression of auxin response pathways results from reduced IAA accumulation in *CYP71Z2*-overexpressing rice.

JA and SA signaling pathways play an important role in broad-spectrum and durable disease resistance of rice. More studies are finding that immunity conferred by SA or JA is independent of IAA resistance signaling in plants, with no correlation reported between suppression of auxin signaling and the activation of SA/JA signaling pathways in resistant rice [12, 49]. Moreover, plant immunity mediated by SA is often accompanied by inhibition of auxin signaling, including down-regulation of auxin-response genes and IAA-amido synthase genes of the *GH3* family [54]. In this study, qRT-PCR analysis showed that the expression of four key genes involved in SA/JA signaling was significantly decreased (Fig. 10), suggesting that SA and JA signaling pathways are inhibited by overexpression of *CYP71Z2* in rice. These results demonstrate that activation of SA or JA signaling pathways is not required for disease resistance mediated by IAA in *CYP71Z2*-overexpressing rice.

The P450 family is the largest protein family in rice and plays an important role in the growth, development and defense responses of this plant. The function of some P450 genes in auxin biosynthesis has been studied in *Arabidopsis*, although so far, similar functionality has not been studied for P450 genes in rice. In this study, the overexpression of *CYP71Z2* in rice increased resistance to *Xoo* PXO99^A^ with suppression of IAA accumulation and IAA response genes, suggesting that the P450 gene *CYP71Z2* takes part in IAA signaling in rice. Moreover, no significant differences in IAA accumulation were detected between *CYP71Z2*-RNAi rice and wild-type, which could be due to residual *CYP71Z2* mRNA or functional redundancy among CYP71Z subfamily proteins. Regardless, these results show that a P450 gene plays a significant role in resistance to pathogen infection in transgenic rice by mediation of the auxin signaling pathway.

## Supporting Information

S1 Fig The construction of transformation vectors for *CYP71Z2* expression pattern. (A) Promoter clone and vectors construction. 1, the amplification of *CYP71Z2* promoter fragment; 3, the double enzyme digestion of P121/PRO plasmids; 2 and 5, DNA Marker DL 2000; 4, DNA Marker λ-*Hin*dIII. (B) Schematic representation of the transformation constructions for *CYP71Z2* expression pattern. RB and LB indicate the right and left T-DNA borders, respectively; NOS indicates the nopaline synthase terminator; NOSP indicates the promoter of the gene encoding nopaline synthetase; *NPTII* indicates the bacterial kanamycin resistance gene (selection marker); Pro indicates the promoter of *CYP71Z2*; *gus* indicates the *E*. *coli β*-glucuronidase gene.
(TIF)

S1 Table Resistance data of *CYP71Z2*-transgenic lines to *Xoo* strain PXO99^A^ at booting stage. (DOC)

S2 Table Gene-specific primers used for qRT-PCR analysis and amplication in this article. (DOC)
