# Supplementary figures and images for: Overexpressing CYP71Z2 Enhances Resistance to Bacterial Blight by Suppressing Auxin Biosynthesis in Rice
Source: PLoS One. 2015 Mar 18;10(3):e0119867. doi: 10.1371/journal.pone.0119867 (PMC4364752; doi:10.1371/journal.pone.0119867)

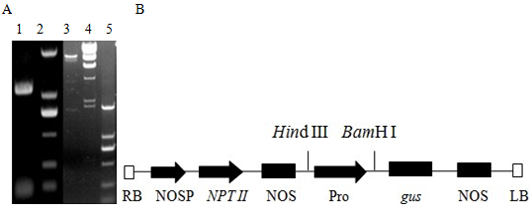

Supplement: S1 Fig — (A) Promoter clone and vectors construction. 1, the amplification of CYP71Z2 promoter fragment; 3, the double enzyme digestion of P121/PRO plasmids; 2 and 5, DNA Marker DL 2000; 4, DNA Marker λ-HindIII. (B) Schematic representation of the transformation constructions for CYP71Z2 expression pattern. RB and LB indicate the right and left T-DNA borders, respectively; NOS indicates the nopaline synthase terminator; NOSP indicates the promoter of the gene encoding nopaline synthetase; NPTII indicates the bacterial kanamycin resistance gene (selection marker); Pro indicates the promoter of CYP71Z2; gus indicates the E. coli β-glucuronidase gene. (TIF) [file pone.0119867.s001.tif]
