# Supplementary material for: Overexpressing CYP71Z2 Enhances Resistance to Bacterial Blight by Suppressing Auxin Biosynthesis in Rice
Source: PLoS One. 2015 Mar 18;10(3):e0119867. doi: 10.1371/journal.pone.0119867 (PMC4364752; doi:10.1371/journal.pone.0119867)
Supplement: S1 Table — (DOC) [file pone.0119867.s002.doc]

**S1_Table Resistance data of *CYP71Z2*-transgenic** **lines to *Xoo* strain PXO99^A^ at booting stage.**

| Rice materials | Relative expression level^a^ | Disease area (%)^b^ | Phenotype^c^ |
| --- | --- | --- | --- |
| **Nipponbare** | 1 | 48.81±6.37 | S |
| **T5 *CYP71Z2*-overexpression lines** | | | |
| OE4 | 8.653±0.482 | 4.31±1.07 | R |
| OE7 | 9.325±0.539 | 4.68±0.82 | R |
| OE11 | 7.416±0.643 | 4.66±0.73 | R |
| OE26 | 9.561±0.752 | 2.69±0.84 | R |
| OE35 | 10.576±0.684 | 3.06±0.52 | R |
| OE51 | 10.438±0.867 | 2.83±0.47 | R |
| **T5 *CYP71Z2*-RNAi lines** | | |  |
| R2 | 0.019±0.013 | 54.36±5.64 | S |
| R3 | 0.036±0.028 | 47.36±5.69 | S |
| R7 | 0.038±0.011 | 50.83±6.56 | S |
| R11 | 0.046±0.041 | 49.37±3.51 | S |
| R15 | 0.083±0.026 | 51.32±5.42 | S |
| **Nipponbare** | 1 | 50.12±6.59 | S |
| **T6 *CYP71Z2*-overexpression lines** | | | |
| OE4 | 10.256±0.681 | 4.13±0.91 | R |
| OE7 | 9.541±0.649 | 4.56±0.43 | R |
| OE26 | 8.164±0.659 | 3.34±0.56 | R |
| OE11 | 7.869±0.851 | 5.36±1.58 | R |
| OE35 | 8.861±1.213 | 4.37±0.64 | R |
| OE51 | 6.843±0.834 | 4.04±0.86 | R |
| **T6 *CYP71Z2*-RNAi lines** | | | |
| R2 | 0.026±0.028 | 56.78±5.67 | S |
| R3 | 0.057±0.031 | 50.39±6.49 | S |
| R7 | 0.068±0.024 | 52.95±4.72 | S |
| R11 | 0.094±0.051 | 45.22±5.29 | S |
| R15 | 0.088±0.029 | 48.63±7.59 | S |
| **Nipponbare** | 1 | 47.37±5.47 | S |
| **T7 *CYP71Z2*-overexpression lines** | | | |
| OE4 | 8.163±0.376 | 1.86±0.56 | R |
| OE7 | 9.578±0.873 | 3.68±0.78 | R |
| OE11 | 10.527±0.826 | 3.16±0.59 | R |
| OE26 | 8.842±0.511 | 4.75±0.93 | R |
| OE35 | 12.351±0.957 | 4.46±0.51 | R |
| OE51 | 11.716±0.967 | 5.31±0.21 | R |
| **T7 *CYP71Z2*-RNAi lines** | | | |
| R2 | 0.026±0.014 | 50.68±6.78 | S |
| R3 | 0.056±0.041 | 51.24±5.62 | S |
| R7 | 0.038±0.025 | 48.46±6.43 | S |
| R11 | 0.047±0.028 | 47.52±4.65 | S |
| R15 | 0.096±0.042 | 53.35±5.18 | S |

^a^ The expression level is relative to *CYP71Z2* expression in the wild-type Nipponbare. The standard deviation of the mean is indicated. ^b^ The disease area is the average of four to five uppermost fully expanded leaves with *Xoo* infected. ^c^R is resistance disease, S is susceptibility disease. Each data represents mean ± standard deviation.
