# Supplementary material for: Overexpressing CYP71Z2 Enhances Resistance to Bacterial Blight by Suppressing Auxin Biosynthesis in Rice
Source: PLoS One. 2015 Mar 18;10(3):e0119867. doi: 10.1371/journal.pone.0119867 (PMC4364752; doi:10.1371/journal.pone.0119867)
Supplement: S2 Table — (DOC) [file pone.0119867.s003.doc]

**S2_Table Gene-specific primers used for qRT-PCR analysis and amplication in this article**

| Primer | Sequence (5’ to 3’) | Genes | Accession Numbers |
| --- | --- | --- | --- |
| *CYP71Z2* Pro-F | cccaagcttGTACAAGGGCTGACAGATGGGT | promoter | NM_001065728 |
| *CYP71Z2* Pro-R | cgcggatccGCCCGTAGGATCGATGTGCTGTA |
| *EF1a*-F | ACAAGCTTGGAGGTATCGAC | *EF1-α* | GQ848072.1 |
| *EF1a*-R | CGCTCGGCCTTGAGCTTGTC |
| *AAO1*-F | CTCGGGTAACATGTGTCGATG | *AAO1* | Os07g0282300 |
| *AAO1*-R | ATATATTAAGGCCCAAATCCT |
| *NIT1*-F | TGCTCCAACTGCTGATTTCTC | *NIT1* | Os02g0635000 |
| *NIT1*-R | TGGATAGTTCTTTCTGCGACA |  |  |
| *IAA1*-F | CACCAAGAGCCGCTCAATGA | *IAA1* | Os01g0178500 |
| *IAA1*-R | ATATCACACGTGGGCGAACAT |
| *IAA14*-F | GCGCACACATGATTGAT | *IAA4* | Os09g0527700 |
| *IAA14*-R | CGCATCTGCAGTGTCAACTA |
| *IAA114*-F | CGCCCTGATGTGTAACA | *IAA14* | Os03g0742800 |
| *IAA114*-R | TTTCACAGCGCCAATAA |
| *IAA120*-F | GGGTCCTGCCGCAAGAG | *IAA20* | Os06g0166500 |
| *IAA120*-R | ATTTCGCCGTCGGAATC |
| *EXPA1*-F | TCGGCTCCATCCAGTCGGTG | *EXPA1* | Os04g0228400 |
| *EXPA1*-R | CGTCGAGGTAGGCGTTGGAC |
| *EXPA5*-F | CGGCGCGTGCTTCGAGGTGC | *EXPA5* | Os02g0744200 |
| *EXPA5*-R | AAGTTGGTGGCCGTGACGAC |
| *EXPA10*-F | CGCGTCAACTGTCTTCGTGAT | *EXPA10* | Os04g0583500 |
| *EXPA10*-R | CACTGATCGGACATCGCCAGC |
| *EXPB3*-F | GAACTTCAGCGCCTCCGACTT | *EXPB3* | [NM_197700.1](http://www.ncbi.nlm.nih.gov/nuccore/NM_197700.1?report=genbank) |
| *EXPB3*-R | AAGCACCGCCATCGTCGTCAG |
| *EXPB4*-F | GGGATGCGGCTCTTGCTACAA | *EXPB4* | Os10g0556100 |
| *EXPB4*-R | GGAGCCACCGGGTAGTAGTTC |
| *EXPB7*-F | CCGGCATCATCGACATCCAGT | *EXPB7* | *Os03g0102700* |
| *EXPB7*-R | CTCCACCAGTATCGCCATGTA |
| *PR1a*-F | AGCTTCAATTAATGGCGAGTT | *PR1a* | AJ278436 |
| *PR1a*-R | GTTGTGCGGGTCCACGAAGTC |
| *PR1b*-F | AGGTATCCAAGCTGGCCAT | *PR1b* | NM_001049624 |
| *PR1b*-R | GAGCCTCACGTAGTCCTGC |
| *AOS2*-F | CAATACGTGTACTGGTCGAATGG | *AOS2* | NM_001055971 |
| *AOS2*-R | AAGGTGTCGTACCGGAGGAA |
| *LOX*-F | GCATCCCCAACAGCACATC | *LOX* | NM_001068734 |
| *LOX*-R | AATAAAGATTTGGGAGTGACATATTGG |
